# The coordination of attention and action in great apes and humans

**DOI:** 10.1098/rstb.2021.0093

**Published:** 2022-09-12

**Authors:** Michael Tomasello

**Affiliations:** ^1^ Duke University, Durham, NC 27708, USA; ^2^ Max Planck Institute for Evolutionary Anthropology, Deutscher Platz 6, 04103 Leipzig, Germany

**Keywords:** collaboration, great apes, shared intentionality

## Abstract

Great apes can discern what others are attending to and even direct others' attention to themselves in flexible ways. But they seemingly do not coordinate their attention with one another recursively—understanding that the other is monitoring their attention just as they are monitoring hers—in acts of joint attention, at least not in the same way as young human children. Similarly, great apes collaborate with partners in many flexible ways, but they seemingly do not coordinate with others to form mutually obligating joint goals and commitments, nor regulate the collaboration via acts of intentional communication, at least not in the same way as young human children. The hypothesis defended here is that it is precisely in their capacities to coordinate attention and action with others—that is, in their capacities for shared intentionality—that humans are most clearly distinguished from other great apes.

This article is part of the theme issue ‘Revisiting the human ‘interaction engine’: comparative approaches to social action coordination’.

## 1. Introduction

From the beginning of the cognitive revolution in psychology, there have been critics arguing that the use of human psychological concepts in the study of animal behaviour is not appropriate. But science marches on, and we have been learning an immense amount about many different animal species by applying human psychological concepts to them and seeing how they fit and how they must be adjusted. Often in this research, there are both researchers who are ‘boosters’, arguing for continuity with humans, and researchers who are ‘scoffers’, arguing that the human concepts must be adjusted significantly to apply to the non-human species of interest. Arguably, this process of pushing and pulling has led both to more and better empirical research, as well as to conceptual clarifications.

Perhaps because they are so close to humans evolutionarily, much of this research has focused on non-human primates, especially in their social cognition. A new frontier in this approach is what the editors of this special issue are calling joint action coordination. Several scholars, myself among them, have argued that humans coordinate and act jointly with others in some unique ways, and this includes the coordination of attention (including in acts of communication). But recently a number of studies have challenged this claim of human uniqueness by finding similar phenomena in non-human great apes. In my opinion, this disagreement between boosters and scoffers again represents a first step toward a deeper understanding of the relevant phenomena for all of us. I will argue here that the things people have shown in action and attention coordination in apes are not totally human-like. But this does not mean that they are not interesting, important, and potentially unique as compared with other mammals and non-ape primates. I expect pushing back in the other direction, and hopefully together we will reach some consensus at some point in the future.

Here I am concerned specifically with how great apes and humans coordinate their attention and actions. I focus first on the key attentional phenomena of joint attention and referential communication, and then on the key action phenomena of joint commitment and partner coordination.

## 2. Coordination of attention

Many primate species engage in *visual co-orientation*, for example, when a loud noise occurs and the whole group looks in that direction. Many primate species also engage in *gaze following* in which one individual looks where another is looking, even behind barriers, indicating in some cases an attempt to see what the other sees. The term *joint attention* is sometimes used to describe such situations (e.g. [1]), but in the human psychology literature, something more is indicated, specifically, an active coordination of attention in which both individuals know together in their common ground (i.e. common knowledge) that they are co-orienting to an external entity. Thus, one individual knows not only that their partner is looking at the same thing they are looking at, but also that this partner knows the same about them: they both know that they both know that they are attending to the same thing: they are sharing attention in a recursively coordinated manner [2,3]. (Just to complete the terminological picture, the term *mutual attention* is typically used for direct eye-to-eye contact not involving an external entity.) This kind of recursive sharing and coordinating of attention to external entities is critically important in human children's cognitive development, including especially in uniquely human processes of gestural and linguistic communication.

### (a) Joint attention

Carpenter and Tomasello [4] attempted to investigate, among other things, the joint attentional interactions of human-raised, language-trained chimpanzees and bonobos with a human experimenter. They used a criterion commonly used in naturalistic studies of human children to determine joint attention: the child (or ape) alternates attention between an object to which she is co-orienting with an adult and the face/eyes of that adult. Using this criterion, they found episodes of joint attention for both ape species as they interacted with their human partner. The problem is that such alternating attention is a very weak criterion, as infants and apes could simply be looking at the object and then checking to see if the adult human is still there, for security, or perhaps they are checking to see if the adult is going to prohibit play with that object. In direct response to this problem, studies with human infants began to look for different outcomes in infants' social interactions with others as a result of joint attentional engagement with them. For example, studies found that after engaging in joint attention with an adult on an object—as opposed to either partner simply onlooking while the other engaged in object play—infants do not expect the adult to be surprised about the presence of that object, but they are surprised if either one of them was previously simply onlooking. They register the experience of a partner only or best when they are in joint attention with them (e.g. [5,6]). Such experimental studies have not been done with great apes.

More recently, Wolf and Tomasello have taken a different approach. In previous studies with both human adults and infants, it was found that watching a video together led subjects to feel socially closer to one another [7,8]. Wolf & Tomasello [9] applied this method to chimpanzees and bonobos as they interacted both with a human and with a conspecific. They found that when the apes watched a video together with a partner, of whatever species, they subsequently preferred to be physically closer to that partner than after a control condition in which they both viewed different videos. Co-orienting to an external entity had an observable effect on their subsequent social behaviour. Whether or not the co-orientation was actually joint attention is an interesting question that was asked by Wolf & Tomasello [10]. As noted above, in joint attention, I know that I am watching the video and that you are watching the video, but I also know that you are aware of my video watching (and me watching your video watching) also. That is what makes it joint: we both know recursively that we both know we are watching the video. Subjects came into a room in which a human was watching a video, and the subject watched the video for a while also. What differed between conditions was whether the human turned and looked at the subject just as the video came on. If they did, one could argue that the subject knew not only that the partner was attending to the video but also was attending to her attention to the video. But this ‘knowing look’ of the partner had no effect on the chimpanzees; they subsequently chose to be equally distant from the partner whether he did or did not look to them as the video started (in the control condition, he looked to the subject at a later moment after the video was off but before the dependent variable was measured). This was in contrast with human children who chose to be closer to the partner if he had looked to them at the key moment as the video began: the key for them was truly shared attention.

Great apes thus follow gaze direction, engage in mutual gazing and know when they are co-orienting with a partner to an external entity, but by a strict definition, they do not engage with one another in joint attention because they do not coordinate their attention with one another recursively on an entity of mutual interest.

### (b) Referential communication

This difference with humans in processes of joint attention may help to explain why great apes do not engage with others in the same kinds of gestural and linguistic communication as humans. Leaving aside language, we may focus on gestural communication, with which great apes are quite proficient. Once again, the issue is not about reading the mind of the other, but about coordinating with it.

In their natural gestural communication, great apes attend to the attention of others—and even attempt to manipulate the attention of others—in ways that other non-human primates do not. Thus, one of the distinguishing characteristics of great ape gestural communication is the use of attention-getters, for example, slapping the ground or poking a conspecific in the back to draw their attention to themselves (e.g. to initiate play)—which monkeys mostly have not been observed to do on a regular basis [11]. Moreover, when using a visually based gestural signal of any kind, apes know that the intended recipient must see the gesture for it to work, and in experiments, they even ‘go around’ in front of a human who has her back turned in order to gesture to her face [12]. But because these attention-getters are aimed at drawing attention to the self and not to an external entity, they do not involve, strictly speaking, acts of reference in which one individual invites another to jointly attend with her to some external referent.

But two sets of researchers have made observations of chimpanzee gestural communication in the wild that they believe represents acts of reference. First, Pika & Mitani [13] report the use of a ‘directed scratch’ produced mainly by high-ranking males to request grooming of specific body areas. The investigators argue against the interpretation that the groomee is simply scratching where something is itching—and the groomer uses that as a cue for a good place to search for parasites—since if this were true all pairs of chimpanzees should perform this behaviour alike, but the gesture was observed much more frequently between high-ranking males. The most plausible interpretation, then, is that the groomee has learned through his grooming interactions with others a ritualized social strategy for directing attention and behaviour to specific places on his body. Second, Hobaiter *et al*. [14] report four observations of chimpanzees (three involving a single mother–child pair on a single occasion) in which a juvenile extended its hand and arm toward ‘a desirable but unobtainable object’. For example, in one observation, a juvenile extended its hand and arm toward a researcher and her equipment, glancing at her mom during the process.

These observations show that great apes can not only direct the attention of others to themselves but also to external entities. They are further supported by observations of chimpanzees ‘pointing’ for humans to things they want. Leavens and colleagues (e.g. [15,16]) have investigated chimpanzee pointing in some depth and found it to be a flexibly used gesture sensitive to the attentional state of the human recipient. In the typical instance, the ape is interacting with the human through mesh caging, and her pointing behaviour involves thrusting the whole hand in the direction of the desired object, with the fingers sticking through the mesh on some occasions. A reasonable interpretation is that this behaviour represents a kind of ritualized reaching to direct the attention of a human to a desired object. Evidence for this ritualized reaching interpretation is the study by van der Goot *et al*. ([17]; see also [18]), who presented chimpanzees with a desirable object next to a human but some distance away. The chimpanzees basically never pointed to the desired object, but instead locomoted over to it and then reached ritualistically through the mesh for it, whereas human infants tested in the same paradigm pointed from a distance using their index finger. ([19], reported what they considered contradictory data from a similar experimental set-up, but they included a host of non-referential gestures such as begging from the human, banging on the cage or spitting toward the human, without singling out pointing or other referential gestures.)

Great apes thus can direct the attention of others to external entities so that they will do the expected thing, whether that be scratching them or fetching them food. But beginning with their earliest pointing behaviour at around one year of age, human infants point not just to direct attention to things they want, but also simply to share attention and interest to external entities. This means, most typically, holding up objects to show them to others or pointing to objects declaratively simply to share attention. Tomasello & Carpenter [20] looked for behaviour of this type in three young, human-raised chimpanzees in their interactions with a human. In extensive naturalistic observations, they did not observe a single instance of the apes making an active attempt to establish joint attention by holding up an object to show it, by pointing to an object declaratively, or in any other way. They also attempted to elicit such behaviour experimentally in situations in which it is shown by human infants, for example, pointing out an object that is doing interesting things but to which the adult is not currently attending, and again they found no attempts to show or point declaratively. Tomonaga *et al*. also looked for evidence of such behaviours in three young chimpanzees as they interacted not with humans but with their mothers, and again they found no evidence for attempting to establish joint attention through declarative gesturing: ‘The infant chimpanzee can follow another's pointing or gaze … [but] does not ‘share’ attention with others’ [21, p. 228]. And this is arguably true also for even language-trained apes, who communicate almost exclusively for imperative purposes ([22]; the examples reported by [23] are basically cases of language-trained apes recognizing and naming a referent associated with the situation, not attempting to share attention declaratively). Human infants, but not great apes, often simply have the goal of sharing attention to objects with others.

The fact that human infants in some cases have as their only goal the sharing of attention with others is especially clear in the experimental studies of Liszkowski *et al*. [24,25], in which infants were not satisfied with adult reactions to their pointing gesture unless the adult both identified the intended referent and shared their enthusiasm for it. The children had as their only goal that the adult share attention and enthusiasm with them. In addition, there are also studies with human infants' requestive pointing showing that even in the case of successful requests (in which they get what they want), they still want the adult to share attention with them to the intended referent. Thus, if the infant requests a toy horse, and the adult responds by holding up the horse and saying ‘Oh, you want the cow? Sorry, I can only give you the horse’, infants are not happy with this response and quite often repeat the request even though it has already succeeded [26]. The point is that the child's requestive gesture is intended not only to obtain a particular object but to do so by getting the adult to jointly attend with her to the intended referent. There is one study with great apes that is somewhat comparable. Halina *et al*. [27] had a human respond to chimpanzees' and bonobos’ pointing to food either by looking at it but not responding (unwilling) or by looking to the wrong object (misunderstanding). Apes did not respond differently in the two conditions, suggesting that their pointing was mainly aimed at getting the adult to look at the food and then do what they wanted her to; joint attention was not a separate goal.

Finally, the same pattern emerges in studies of the comprehension of declarative or informative pointing in human infants and great apes. If food is hidden in one of two buckets and a human then points to one of the buckets, apes seemingly do not comprehend (see [28], for a review). Apes sometimes even follow the human's pointing and look to the bucket, but then they do not make the seemingly straightforward inference that the human is directing their attention there because he thinks it is somehow relevant to their current search for the food. They do not make this relevance inference because, in one interpretation, it requires them to coordinate their own mental state with that of the human recursively. Specifically, they need to infer something like ‘he *intends* that I *know* where the food is'. This embedding of mental states within one another recursively is not something, apparently, that comes naturally to great apes.

There are several studies suggesting some ape skills in this task. But in virtually all of these studies the ‘pointing’ is very close to touching (10 cm from target or less). Thus, in a review paper, Miklósi & Soproni [29] found that all species of great ape perform poorly when the distance of the pointing gesture from the target is 20 cm away or greater (see also [30]). A reasonable possibility is that the apes see the close-pointing as something like reaching or a preparation for touching it. (This same analysis applies to the study reported in [31], in which pointing occurred from 2 to 10 cm away from the target.) The one exception to this pattern is the study of Mulcahy & Call [32]. They report a study in which chimpanzees apparently succeeded in this task, but the set-up was different. In this case, the human was located between buckets and turned and faced a bucket as he pointed to it, which meant that the ape might have just been coming to see his face, similar to their behaviour in the study of Liebal *et al*. [12]. Moreover, in the study of Herrmann & Tomasello [33], the two containers were in basically the same location as in this study (both studies were conducted in the same space in the same facility with many of the same chimpanzee subjects), but the experimenter stood back a bit and pointed without turning her body. In this case, the chimpanzees failed.

If one defines reference as directing the attention of another to an external entity, then one could say that apes engage in acts of reference. However, if one defines reference as one individual inviting another to share attention with her to a common referent—thus coordinating their attention—then reference may be confined to humans. In either case, there is a demonstrable difference between great ape and human acts of gestural reference via such things as showing and pointing in that humans (i) are motivated to do so declaratively simply to share interest and attention, (ii) work to make sure that their recipient's attention matches theirs even if they have already received what they want, and (iii) embed mental states within one another recursively in comprehension.

### (c) Conclusion

Great apes know a lot about the attention of others: they know what others are attending to and can in some cases manipulate that through gestural communication. But what humans do, from early in ontogeny, is to coordinate their attention with others in acts of joint attention: they understand that just as they are attending to the other's attention the other is attending to their attention, and they actively manage this coordination of attention in acts of referential communication, including acts that are aimed only a joint attention as a goal. Humans have both the skill and the motivation to coordinate and share attention with others recursively.

## 3. Coordination of action

The simplest and most pervasive form of collaboration in great apes and other primates is the formation of coalitions to compete with others in the group for resources and dominance status. Such coalitions are crucial in the social structure of the group in many ways, but from a cognitive point of view they do not seem to involve much complex coordination or communication between individuals acting as partners, beyond simply fighting side by side against a common opponent.

The most complex coordination of action in non-human primates is almost certainly the group hunting of chimpanzees. In many but not all chimpanzee populations, individuals hunt together in a small group for monkeys. The basic idea is that because monkeys are so quick and agile in the trees, the chimpanzees must surround one in order to capture it. In formulating its plan of action, the individual chimpanzee takes into account not only the actions of the monkey, but also the actions, and even intentions, of the other hunters. There is thus no question that participating in a group hunt requires of individual chimpanzees many complex cognitive skills. The question is how individuals engage and coordinate with one another as they pursue their prey—and how similar this process is to that of human collaboration. We look first at how chimpanzees and humans initiate collaborative activities and then how they coordinate those activities with partners.

### (a) Joint goals and commitments

Initiating a collaborative activity is not as simple as it seems. Duguid *et al*. [34] confronted pairs of chimpanzees with a collaborative problem modelled after the Stag Hunt in game theory. Each chimpanzee was feeding on a low value food (raisins) when a high-value food (bananas) appeared some metres away. A spring-loaded, locking door on the raisins ensured that going for the bananas meant forsaking the raisins. What happened for almost all pairs on almost all trials was that one individual simply took off for the bananas first, incurring a significant risk. The other individual then just followed. This leader–follower strategy worked fine as long as everything was out in the open. But when experimenters placed a barrier so that the apes could not see one another easily (they could do so only if they raised up their bodies to look over), performance went down significantly. They still succeeded sometimes because both individuals just went for the bananas straightaway, expecting or hoping the other would do the same, but they never communicated before they abandoned their raisins, even though they could easily have done so by making noise or gesturing over the barrier (which human children often did in the same situation). This study provides a plausible model for how chimpanzees' group hunting of monkeys begins, as one individual takes the risk and others follow. Neither chimpanzee hunters nor chimpanzee subjects in this experiment arrange ahead of time a joint goal that they only then pursue together, as human children often do.

Warneken *et al*. [35] addressed the question of whether chimpanzees form joint goals with others by looking at how they respond to disruptions to collaborative activities. They tested both young human-raised chimpanzees and 18-month-old human infants in a series of four collaborative tasks with a human adult, such things as obtaining a toy by each operating one side of an apparatus. Then, the adult simply stopped playing her role for no reason. The children were not happy about this and did various things to attempt to re-engage their partner. And children do not attempt to re-engage a partner only because he is necessary for the game: he is more than a useful social tool; he is a collaborative partner. Thus, Warneken *et al*. [36] found that human children also attempt to re-engage recalcitrant partners even when the game could be played in the same basic way alone. This experiment has not been done with great apes. And young children can coordinate with one another as well, from around 2 years of age, as shown by Brownell & Carriger [37], among others.

By contrast, in the Warneken *et al*. [35] study, when the same games were played with the chimpanzees, they did not attempt to re-engage the experimenter; they simply ignored the uncooperative partner and tried to find ways to achieve the goal on their own. However, in some more recent studies with bonobos, researchers have observed more human-like re-engagement responses to disruptions to collaboration. Pika & Zuberbühler [38] observed bonobos playing together with humans with objects. They report four social games that seemed to them to be collaborative—perhaps suggesting a joint goal—mainly because when the human ceased playing, the bonobo did something to prod her to continue. MacLean & Hare [39] also observed captive bonobos in interaction with humans and found that they preferred engaging with objects in interaction with humans rather than alone, and that, again, they would actively seek to re-engage a recalcitrant partner. Interestingly, when two bonobos were given an object, each preferred to play with it alone rather than to play collaboratively, and they did not attempt to re-engage recalcitrant partners at all. So somehow apes need an especially competent and motivated collaborative partner in a way that human children do not (see, e.g. [37]).

But beyond joint goals are joint commitments. When individuals make a joint commitment to collaborate, they implicitly agree to stick with it until both get their rewards even in the face of temptations to defect. One simple example is an experiment by Greenberg *et al*. [40], who had pairs of chimpanzees work together in a collaborative task, but for one individual the reward, surprisingly, became available midway through. In almost every instance, the lucky chimpanzee took her reward and left the scene; the other chimpanzee was a useful social tool, but not a partner with whom one had a joint commitment to pursue rewards together to the end. By contrast, in the same study with children, the lucky child delayed consumption of her own reward and persevered until the other got hers [41]. Such a joint commitment between partners suggests that, unlike the apes who were using their partners as a social tool, the children had committed from the beginning that ‘we’ get the rewards together, and they did whatever was necessary to realize that joint goal.

Other researchers have studied bonobos interacting with one another and argued that they, and perhaps other great apes, do indeed form joint commitments to do things together. Genty *et al*. [42] report seven instances, based on detailed observations, of the intricate ways in which bonobos coordinate their social interactions, and the ways they resume them after interruption. For example, two captive bonobos were grooming when a noise occurred some distance away. They both went over to investigate, but then, being satisfied, they resumed their grooming, coordinating their gaze and using gestures to get back into the same grooming roles (even grooming the same body parts) as before. The authors comment: ‘The resumption of the activity with the same partner and at the same location, after being interrupted by an external event, having relocated and being physically separated, and the reengagement via communicative signals, suggests the possibility that Lisa and Vic are both committed to grooming each other at a specific location until both are ready to terminate the activity’ [42, p. 382]. This and similar observations may reflect something like a joint commitment, but they also may reflect simply high motivation to resume engaging in a rewarding social activity; that is, they may reflect a preference not a commitment, as commitments are typically diagnosed when partners sacrifice to maintain the collaboration.

Heesen *et al*. [43] introduced experimentally planned interruptions with bonobos. Human experimenters either called one individual bonobo's name or created noises suggestive of imminent feeding. They did this either when that individual was socially engaged with a partner in grooming or else when it was engaged in solitary grooming or play. After the interruption, the bonobos who were engaged in social grooming were more likely to return to that activity than were the bonobos who were engaged in solitary grooming or play. When resuming the social activities, there was sometimes gestural and/or vocal communication. The authors take this pattern of results to suggest that the social partners had a joint commitment to groom together, re-engaging after a disruption. But, again, it is just as natural to interpret these results as suggesting not a normative commitment but a simple preference for social over solitary activity. This simpler interpretation is viable because many studies with human children employ stricter criteria. In these studies (see [44], for a review), the comparison is not between a child engaged in a solitary activity versus a joint or group activity, as in the bonobo studies, but rather between two group activities, one of which was initiated by one partner simply joining another unbidden and the other of which was initiated by an explicit joint commitment created through a communicative act of some kind. In both cases, the children are engaging with others socially, but in one case, an explicit joint commitment has been made. Then, what is measured is not simply a preference for returning to the activity, but some recognition of the normative bond or obligation that the joint commitment has created. Thus, when a partner seemingly breaks a joint commitment, children actively protest [45], unless the partner excuses himself before breaking the commitment in which case all is forgiven [46]. When children themselves feel the need to break a joint commitment, they actively excuse themselves, ask permission or take leave [47]—or else they show guilt and remorse [48].

The point is that joint commitments engender in humans a sense of obligation to the partner, which can be observed in such things as persistence until both reach a goal, protest against breaches, apologies for one's own breaches, request for permission to breach, taking leave before breaching and feelings of guilt after a breach. Until we see signs of such behaviour in great apes in their collaborative activities—which, admittedly, will be difficult if they are not communicating via language—it is more plausible to interpret their behaviour as expressing a preference rather than a commitment.

### (b) Partner coordination

Chimpanzees know that in some situations they need a partner to succeed. Thus, male chimpanzees in the wild recognize that to succeed in pursuing a monkey, they must have other males with them; chimpanzees travelling alone almost never initiate a chase. In an experiment, Melis *et al*. [49] found that chimpanzees actively recruited a needed collaborator by actually opening a door for them, and, further, they recruited the more effective of two partners on the basis of their past experience with them. Nevertheless, chimpanzees would rather obtain food on their own if they can. When individuals are presented simultaneously with two equally rewarding options—pull in food alone (on one side of the cage) or pull in food with a partner (on the other side of the cage; partner in adjacent cage)—they choose each equally often. By contrast, human children prefer the collaborative option [50,51].

Beyond just knowing that one needs a partner, skillful collaboration requires partners to pay attention to one another's actions and, possibly, to one another's decisions. This often requires understanding what the partner is intending to do, and how her actions affect what one should do oneself. Fletcher *et al*. [52] presented pairs of chimpanzees with a task requiring two active and complementary roles. In a baseline condition, the target subject was asked to play Role B, as we may call it, with no previous experience. In the experimental condition, the target subject had previously played, with a different partner, Role A. The finding was that the subjects who had previously played Role A were subsequently no more proficient in Role B than were the subjects that had never played Role A. Chimpanzees did not learn anything about Role B from having played the reciprocal role previously. By contrast, human children in the same experimental situation did learn something about the reciprocal role from similar past experiences. But the task in this experiment was complex and may have been a special challenge to learn vicariously from the other role.

Melis & Tomasello [53] therefore presented chimpanzees with a simpler and more natural collaborative food-retrieval task requiring complementary roles and tested subjects' ability to help their partner perform her role. For each role, subjects required a different tool, and the tools were not interchangeable. Experimenters gave one individual in a dyad both tools and measured her willingness to transfer a tool to their partner, as well as which tool (correct versus incorrect) she transferred. Most subjects helped their partner and transferred to him the tool that he needed. Thus, in a relatively simple task, chimpanzees do indeed know which particular action their collaborative partner needs to perform (see also [54]). Grüneisen *et al.* [55] provided a complementary finding. Pairs of chimpanzees and bonobos engaged in a collaboration task from opposite sides of an apparatus (from different rooms). A strategically placed barrier meant that partners could only see one another's actions if they moved out from behind the barrier. Seeing the partner's actions facilitated coordination, and thereby the partners' joint success. Both ape species went to some efforts to make sure that their actions were visible to their partner (which they did less often in a competition condition in which it was to their benefit to hide their actions). Thus, across these two studies, the apes seemed to understand the role of a collaborative partner to the degree that they knew both the tool she needed and what she needed to see to perform her role in the best way for their collaborative success.

But beyond even this understanding of a partner's actions and perceptions, coordination (in the game theory sense of the term) requires monitoring and adjusting to a partner's decisions. Thus, Duguid *et al*. [56] tested pairs of chimpanzees in a so-called ‘pure coordination’ game in which they had available multiple possible rewards simultaneously; to succeed they had to both decide, more or less simultaneously, to choose the same option. Nevertheless, the apes had great difficulty coordinating on a box. After enough trials, a given pair might settle on one particular box on every trial and thereby become successful. But then, individuals from different successful pairs were put together, and they took just as long to settle on a particular box with this new partner. What they were learning was to choose a particular box. By contrast, human children in the same situation coordinated their decisions very quickly, and with each new partner they coordinated decisions ever more quickly, presumably because they understood that what mattered was not choosing a particular box but coordinating on the same box, whichever box that might be. They often facilitated their coordination by communicating about their impending decision. Coordinating with a partner to obtain food—when multiple options are simultaneously available—is something that comes much more readily to human children than to great apes.

Great apes thus have some skills in coordinating behaviour with a partner, but what they seem to lack is the ability to coordinate decisions ahead of time, at least partly because they are not inclined to communicate with an ape partner during collaboration [57]. In general, when apes collaborate with one another, there is very little communication to coordinate the process of joint decision making. Thus, Melis *et al*. [58] presented pairs of chimpanzees with a choice between two cooperative tasks, one each in adjacent rooms, with failure to work together on either of them resulting in no payoff for either partner. The dominant partner preferred the option with unequal payoffs because she could dominate the larger payoff, whereas the subordinate preferred the option with equal payoffs because then she could get more. Quite often an individual went to the doorway between rooms and stared at the other, but neither individual made any communicative attempt to exhort or cajole their partner into choosing the option she preferred. Further in this direction, Bullinger *et al*. [59] created a situation in which chimpanzees could help a partner play her role in a mutually beneficial food-retrieval task either by transferring a needed tool to her (helping condition) or by visually or acoustically communicating the hiding location of the needed tool (communication condition). Overall, chimpanzees readily helped their partner by delivering the needed tool, but none of them communicated the hiding location of the tool to their partner reliably across trials.

Melis & Tomasello [60] thought that perhaps, with appropriate experiences, chimpanzees might be able to communicate in order to coordinate. They tested pairs of chimpanzees in the collaborative task of Melis & Tomasello [53], in which they had all previously participated, so that both members of a pair already knew that to extract rewards from an apparatus each of them needed a particular tool. Then, in this study, the tools were in one of two boxes in one chimpanzee's cage, but she could not see them and did not know which box contained them. The partner, in an adjacent cage, could see the tools and so could potentially communicate their location to the partner. The knowledgeable individuals never used any overt gestures or vocalizations to single out the correct box. But they did quite often position themselves behind or very close to it, sometimes even touching or looking at it. But partners nevertheless chose boxes randomly overall. Across many trials, the chimpanzees did learn to coordinate by one individual approaching the correct box and the other coming over to that box. But still there were no overt acts of intentional communication such as, for example, slapping the top of the box or reaching towards it ritualistically (acts of which they are perfectly capable). In combination with the observations of an almost total lack of communication in other collaborative contexts, this suggests, at the very least, that communicating in order to coordinate does not come naturally and easily to chimpanzees.

### (c) Conclusion

Great apes are thus able to coordinate with a partner to some degree, knowing that they need a partner, knowing their partner's goal and knowing that their partner needs to see their actions. But the degree to which they can coordinate is severely limited by their limited skills of cooperative communication. Indeed, Tomasello [61] argues that humans' species-unique skills of cooperative communication—using either gestures or linguistic conventions to inform others about referents helpfully—first arose in the context of collaborative activities where helping one's partner play her role (e.g. by informing her of relevant things) facilitates collaborative success.

## 4. Shared intentionality and mental coordination

Humans are cognitively different from great apes not because they are better at reading minds but because they mentally coordinate with others in unique ways. One possible evolutionarily story is that at some point humans came under ecological pressure to forage collaboratively with others or starve, and this also involved partner choice so that there was social selection for good collaborators [62]. Early humans adapted to these new ecological pressures by evolving skills and motivations of shared intentionality that enabled them to form with one another joint goals and commitments and to coordinate attention with one another more sensitively in their collaborations, including through the use of various forms of cooperative and conventional communication. These new forms of collaboration restructured human cognition by requiring the recursive coordination of mental perspectives.

These new skills of shared intentionality not only facilitated collaboration, but also changed other important aspects of human cognition. Thus, because great apes do not engage in human-like joint attention, they do not understand in a human-like way the notion of perspective, which explains why they do not understand in a human-like way the notion of belief as a coordination of different perspectives (individuals' particular perspectives and an objective perspective) or fairness as a coordination of the different interests of the different parties involved [63]. Skills of mental coordination are also necessary for cooperative and conventional linguistic communication, as well as for teaching and therefore cumulative culture [64,65]. It is thus at least possible that non-human primates engage in sophisticated mindreading, but because they cannot coordinate their own mental states with those of others in human-like ways, their mentalizing is not the same as humans’.

## Data Availability

This article has no additional data.
